# Different Rates of the *SLC26A4*-Related Hearing Loss in Two Indigenous Peoples of Southern Siberia (Russia)

**DOI:** 10.3390/diagnostics11122378

**Published:** 2021-12-17

**Authors:** Valeriia Yu. Danilchenko, Marina V. Zytsar, Ekaterina A. Maslova, Marita S. Bady-Khoo, Nikolay A. Barashkov, Igor V. Morozov, Alexander A. Bondar, Olga L. Posukh

**Affiliations:** 1 Federal Research Center Institute of Cytology and Genetics, Siberian Branch of the Russian Academy of Sciences, 630090 Novosibirsk, Russia; danilchenko_valeri@mail.ru (V.Y.D.); zytzar@bionet.nsc.ru (M.V.Z.); maslova@bionet.nsc.ru (E.A.M.); 2 Novosibirsk State University, 630090 Novosibirsk, Russia; Mor@niboch.nsc.ru; 3 Perinatal Center of the Republic of Tyva, 667000 Kyzyl, Russia; marita.badyhoo@mail.ru; 4 Yakut Scientific Centre of Complex Medical Problems, 677019 Yakutsk, Russia; barashkov2004@mail.ru; 5 M.K. Ammosov North-Eastern Federal University, 677027 Yakutsk, Russia; 6 Institute of Chemical Biology and Fundamental Medicine, Siberian Branch of the Russian Academy of Sciences, 630090 Novosibirsk, Russia; alex.bondar@mail.ru

**Keywords:** hearing loss, genetic diagnosis, *SLC26A4*, DFNB4, Tuvinians, Altaians, Southern Siberia, Russia

## Abstract

Hereditary hearing loss (HL) is known to be highly locus/allelic heterogeneous, and the prevalence of different HL forms significantly varies among populations worldwide. Investigation of region-specific landscapes of hereditary HL is important for local healthcare and medical genetic services. Mutations in the *SLC26A4* gene leading to nonsyndromic recessive deafness (DFNB4) and Pendred syndrome are common genetic causes of hereditary HL, at least in some Asian populations. We present for the first time the results of a thorough analysis of the *SLC26A4* gene by Sanger sequencing in the large cohorts of patients with HL of unknown etiology belonging to two neighboring indigenous Turkic-speaking Siberian peoples (Tuvinians and Altaians). A definite genetic diagnosis based on the presence of biallelic *SLC26A4* mutations was established for 28.2% (62/220) of all enrolled Tuvinian patients vs. 4.3% (4/93) of Altaian patients. The rate of the *SLC26A4*-related HL in Tuvinian patients appeared to be one of the highest among populations worldwide. The *SLC26A4* mutational spectrum was characterized by the presence of Asian-specific mutations c.919-2A>G and c.2027T>A (p.Leu676Gln), predominantly found in Tuvinian patients, and c.2168A>G (p.His723Arg), which was only detected in Altaian patients. In addition, a novel pathogenic variant c.1545T>G (p.Phe515Leu) was found with high frequency in Tuvinian patients. Overall, based on the findings of this study and our previous research, we were able to uncover the genetic causes of HL in 50.5% of Tuvinian patients and 34.5% of Altaian patients.

## 1. Introduction

Hearing loss (HL) is one of the most common sensory disorders affecting over 5% of the world’s population [1]. Approximately half of all HL cases are attributed to genetic causes [2]. Hereditary HL includes many different syndromes with HL as one of the clinical symptoms and more common nonsyndromic forms. Over 160 nuclear genes are causally implicated in nonsyndromic HL with different types of inheritance: autosomal dominant—DFNA, autosomal recessive—DFNB, or X-linked—DFNX [3]. In addition, some mutations in mitochondrial DNA are also associated with HL [4]. Mutations in the *GJB2* gene (13q12.11, OMIM 121011) encoding transmembrane protein connexin 26 result in the nonsyndromic autosomal recessive deafness 1A (DFNB1A, OMIM 220290), which is one of the most common forms of HL in many populations, at least of Caucasian descent [5]. Testing of *GJB2* mutations is efficient for establishing a genetic diagnosis in many HL cases. However, the causes of HL in a large number of patients often remain unknown because of high locus/allelic heterogeneity and varying prevalence of hereditary HL in different populations.

Mutations in the *SLC26A4* gene (Solute carrier family 26, member 4/pendrin, 7q22.3, OMIM 605646) are considered to be the second commonest cause of hereditary HL in most world populations, at least in East Asia (Japan, Korea, China) and Mongolia [6,7,8,9,10,11,12,13]. The *SLC26A4* gene encodes pendrin, a protein belonging to the SLC26 anion transporter family, which is mostly expressed in tissues of the inner ear, thyroid, and kidneys and is involved in the transport of various anions [14,15]. In the inner ear, pendrin maintains anionic composition of endolymph by mediating Cl^−^/HCO_3_-exchange [16]. Mutations in the *SLC26A4* gene cause non-syndromic recessive deafness (DFNB4, OMIM 600791) and Pendred syndrome (PDS, OMIM 274600), which combines sensorineural HL and goiter. A prominent clinical characteristic of inner ear in the *SLC26A4*-related HL is the enlarged vestibular aqueduct (EVA) and other malformations of inner ear structures detected by computed tomography (CT) or magnetic resonance imaging (MRI) [17]. Two radiologic criteria are used to establish EVA: a historically earliest and most commonly used “Valvassori criteria” (a midpoint diameter of the vestibular aqueduct >1.5 mm) [18] and relatively recent “Cincinnati criteria” (a midpoint diameter ≥1.0 mm or an operculum diameter ≥2.0 mm) [19,20]. Murine model studies revealed pendrin to be responsible for maintenance of endocochlear potential and fluid homeostasis in the cochlea. The deficiency or dysfunction of pendrin causes endolymphatic hydrops with enlargement of the vestibular aqueduct and endolymphatic sac, as well as other abnormalities of the inner ear structures, being presumably a consequence of defects in anion and fluid transport [21]. However, the pathogenesis of EVA may also be attributed to other mechanisms since not all patients with detected EVA have the *SLC26A4*-related HL [22].

To date, more than 500 variants in the *SLC26A4* gene associated with a wide range of HL phenotypes have been reported (Human Gene Mutation Database: http://www.hgmd.cf.ac.uk/ac/index.php (accessed on 1 November 2021) [23]. Screening for *SLC26A4* mutations has become an important part of molecular genetic testing for HL, especially for patients with detected EVA. Nevertheless, despite numerous studies, the pathogenic contribution of *SLC26A4* to HL in different populations remains to be accurately estimated. First, this is due to the heterogeneity of the examined cohorts of patients in different studies, which varied in size and phenotypic characteristics of enrolled patients (pediatric or adult samples, cochlear implantees, patients with nonsyndromic sensorineural HL (NSHL), patients with diagnosed EVA or Pendred syndrome). Second, methods for the *SLC26A4* analysis varied from a target screening of only the most prevalent *SLC26A4* mutations to a thorough study of the *SLC26A4* coding and adjoined regions or the whole *SLC26A4* sequence by Sanger sequencing or NGS technology.

Different proportions of patients having biallelic *SLC26A4* mutations were revealed in a relatively limited number of large NSHL studies performed without preselection of patients with EVA or Pendred syndrome: 3.5% of sib pairs from the UK Caucasian child population [24], 0.9% of Czech patients [25], 2.9% of Brazilian patients [26], 6.3% (0–8.3%) of patients from different regions of Iran [27], 7.2% of patients from Pakistan [28], 3.5% of patients from southern India [29], 1.1% of Korean patients [6], 4.6% of the Vietnamese pediatric population [30], 1–1.5% of Mongolian patients [6,12], up to 15.3% of patients from different regions of mainland China [31,32,33,34], and 5.8% of Taiwanese patients [13]. A significantly higher proportion of biallelic *SLC26A4* mutations was found in the studies on cohorts of patients who were pre-screened for EVA, reaching 65–95% in Asian cohorts and approximately one-fourth of patients with nonsyndromic EVA in Caucasian cohorts, which is probably influenced by the different ethnicities of patients and an increased sensitivity of sequencing techniques [7,10,22,35,36,37].

In numerous studies, the *SLC26A4* mutation spectrum and prevalence were found to be very diverse around the world and were considered to be ethnic-specific since some ethnic groups appeared to have different mutational hotspots, although, so far, there are significantly fewer supporting data in comparison with the *GJB2* gene [6,11,38,39]. Meta-analysis performed by Lu et al. (2015) revealed 26 out of 272 different *SLC26A4* mutations that were in the top 10% of mutation rates in patients with HL worldwide. Among them, c.919-2A>G was the highest frequency *SLC26A4* mutation (62.4%) followed by c.2168A>G (p.His723Arg) (26.1%). Various sets of the *SLC26A4* mutations with frequencies of more than 5% were found only either in Asia or in Europe [39]. It is now evident that the *SLC26A4* mutation spectrum found in Asian populations is quite different from that in populations of Caucasian ancestry [11].

The concentration of the *SLC26A4*-related HL in a particular population or region is probably influenced by a certain population genetic structure and factors of population dynamics as were shown for some other forms of hereditary HL. Investigation of region-specific landscapes of the *SLC26A4*-related HL is important for local healthcare and medical genetic services.

Siberia, a large (over 13.1 million square kilometers) geographical region of the Russian Federation with a population of approximately 36 million in total, is a multiethnic region where, along with numerous Russians, live various indigenous Siberian peoples. Tuvinians (Tuvans) and Altaians, representing two indigenous Turkic-speaking peoples, live in the Republic of Tyva and the Republic of Altai, respectively, bordering each other in Southern Siberia. Both republics also border Mongolia in the south, and the Altai Republic borders China (in the south) and Kazakhstan (in the southwest). Tuvinians, about 250,000 people in total, according to the Russian Census of 2010, live mainly in the Tyva Republic. Besides the Tyva Republic, relatively small groups of Tuvinians also live in the northern part of Mongolia and in the Xinjiang Uygur Autonomous Region of China [40,41]. Tuvinians are one of the most ancient Turkic-speaking peoples inhabiting Central Asia and the Sayan-Altai region. Prolonged relations with residents of neighboring regions (Turkic-, Mongolic-, Ket-, and Samoyedic-speaking tribes) had a significant impact on the formation of the Tuvinian population [42,43]. The Altaians, about 70,000 people in total, according to the Russian Census of 2010, originated from several ancient Turkic-speaking tribes [44]. The archaeological, linguistic, anthropological, and historical evidence indicates similarities in the ethnogenesis of both Turkic-speaking Tuvinians and Altaians.

During our previous molecular genetic studies of the hereditary HL in Tuvinian and Altaian deaf patients, a genetic diagnosis based on the thorough testing for the *GJB2* gene and the target screening of several mutations in other HL-associated genes, was established in many HL cases [45,46,47,48,49,50,51]. Nevertheless, the causes of HL in a significant number of patients remained unknown.

Pathogenic variants in the *SLC26A4* gene are considered as a common cause of HL among many Asian populations; thus, the involvement of *SLC26A4* in the etiology of HL in Tuvinian and Altaian patients living in Southern Siberia (Russia) seems to be quite expected. In this regard, the aim of this work was to evaluate for the first time the *SLC26A4* pathogenic contribution to HL in Tuvinian and Altaian patients.

## 2. Materials and Methods

### 2.1. Study Subjects

#### 2.1.1. Patients

The ethnically matched cohort of patients with HL of unknown etiology from Southern Siberia (Russia) included 170 Tuvinians (the Tyva Republic) and 62 Altaians (the Altai Republic). Analysis of pedigrees and family histories revealed that the group of examined Tuvinian patients consisted of 57 familial (two or more affected family members) and 111 single/sporadic (the only affected individual in family) HL cases while the group of Altaian patients included 36 familial and 26 single/sporadic HL cases. These patients were selected from the main groups of Tuvinian (*n* = 220) and Altaian (*n* = 93) patients and represent individuals in whom the causes of HL remained unknown after the thorough testing for the *GJB2* gene [45,48,49,51] and the target screening of several mutations in other genes (*MT-RNR1, MT-TS1, OTOF, RAI1)* [46,47,50]. Genomic DNA samples of Tuvinian patients were collected from 2010 to 2018, and DNA samples of Altaian patients were collected from 2001 to 2003 with the subsequent addition of samples in 2012.

The hearing status of patients was evaluated by otoscopic and pure-tone audiometry examinations at different times in the specialized audiological services located in the town of Kyzyl (the Tyva Republic) and the town of Gorno-Altaiisk (the Altai Republic). The severity of HL was defined as mild (25–40 dB), moderate (41–70 dB), severe (71–90 dB), or profound (above 90 dB). The majority of examined Tuvinian patients (164 individuals) had congenital or early onset severe-to-profound HL and six patients had moderate HL. Among Altaian patients, 30 individuals had severe-to-profound HL, 18 individuals had moderate HL, and for 14 Altaian patients the severity of HL was not determined. Other concomitant information was collected from local unspecialized medical services and by direct interview with the patients and their relatives. The CT scan of temporal bones in Tuvinian patients with biallelic *SLC26A4* mutations was performed in the Department of Diagnostic Radiology of the Republican Hospital No. 1 (Kyzyl, the Tyva Republic, Russia). Unfortunately, the examination of patients for thyroid dysfunction and/or a goiter using a perchlorate discharge test and a thyroid ultrasound was not available.

#### 2.1.2. Control Samples

The control samples were represented by 157 unrelated Tuvinians and 141 unrelated Altaians from different regions of the Tyva Republic and the Altai Republic, respectively. None of them were registered by audiological services and had complained of hearing impairment.

#### 2.1.3. Ethics Statement

Written informed consent was obtained from all individuals or their legal guardians before they participated in the study. The study was conducted in accordance with the Declaration of Helsinki, and the protocol was approved by the Bioethics Commission at the Institute of Cytology and Genetics SB RAS, Novosibirsk, Russia (Protocol No. 9, 24 April 2012).

### 2.2. Molecular Analysis

Genomic DNA was isolated from the buffy coat fraction of blood by a standard phenol-chloroform extraction method.

#### 2.2.1. Mutation Analysis of the *SLC26A4* Gene

The *SLC26A4* gene sequence encompassing all 21 exons with flanking regions was analyzed by Sanger sequencing. Primer pairs designed to amplify corresponding PCR products and also used for Sanger sequencing are summarized in Supplementary Table S1. The PCR products were purified by sorption on Agencourt Ampure XP (Beckman Coulter, Indianapolis, IN, USA) and subjected to Sanger sequencing using a BigDye Terminator V.3.1 Cycle Sequencing Kit (Applied Biosystems, Waltham, MA, USA) with subsequent unincorporated dye removal by gel filtration on the Sephadex G-50 (GE Healthcare, Chicago, IL, USA). Sanger products were analyzed on an ABI 3130XL Genetic Analyzer (Applied Biosystems/Life Technologies, USA) in the SB RAS Genomics Core Facility (Institute of Chemical Biology and Fundamental Medicine SB RAS, Novosibirsk, Russia). DNA sequence variations were identified by comparison with the *SLC26A4* gene reference sequences: NC_000007.13 (https://www.ncbi.nlm.nih.gov/nuccore/NC_000007.13/ (accessed on 1 November 2021) and NC_000007.14 (https://www.ncbi.nlm.nih.gov/nuccore/NC_000007.14/ (accessed on 1 November 2021).

#### 2.2.2. Screening of Pathogenic *SLC26A4* Variants in Control Samples

Screening of variants c.170C>A (exon 3), c.919-2A>G (intronic region between exons 7 and 8), c.1545T>G (exon 14), and c.2168A>G (exon 19) in control samples was performed by PCR-RFLP assays using primer pairs designed to amplify corresponding PCR products and restriction enzymes *Tru9 I*, *Hpa II*, *Pce I, Rsr2 I*, respectively (Supplementary Table S1). Screening of variants c.2027T>A (exon 17) and c.2034+1G>A (intronic region between exons 17 and 18) in control samples was performed by Sanger sequencing.

### 2.3. Bioinformatics Tools

#### 2.3.1. Bioinformatics Prediction Tools

Functional effect of c.1545T>G (p.Phe515Leu) variant was predicted using PolyPhen-2 (http://genetics.bwh.harvard.edu/pph2), PROVEAN (http://provean.jcvi.org), MutationTaster (http://www.mutationtaster.org/), FATHMM (http://fathmm.biocompute.org.uk/), MutationAssessor (http://mutationassessor.org/), Align-GVGD (http://agvgd.hci.utah.edu/), MutPred2 (http://mutpred.mutdb.org/), Condel (https://bbglab.irbbarcelona.org/fannsdb/), SNPs & GO (https://snps-and-go.biocomp.unibo.it/snps-and-go/), CADD (https://cadd.gs.washington.edu/), SIFT (https://sift.bii.a-star.edu.sg/) (Supplementary Table S2).

#### 2.3.2. 3D Modeling of Pendrin Molecule Structure

The three-dimensional (3D) molecule structure of the wild-type and mutant p.Phe515Leu type of pendrin protein was predicted by the I-Tasser program (https://zhanglab.ccmb.med.umich.edu/I-TASSER/) [52,53,54] and was visualized by Swiss-PdbViewer v.4.1.0 (http://www.expasy.org/spdbv/) [55].

### 2.4. Statistical Methods

Two-tailed Fisher’s exact test with a significance level of *p* < 0.05 was applied to compare allele frequencies between patients and controls.

## 3. Results

### 3.1. SLC26A4 Genotypes of Patients

Analysis of the *SLC26A4* gene was performed in ethnically matched cohorts of patients (170 Tuvinians and 62 Altaians) with HL of unknown etiology. Sequential analysis of the *SLC26A4* gene fragments by Sanger sequencing in a particular patient was continued until two recessive pathogenic *SLC26A4* variants were detected and, therefore, diagnosis could be made. The *SLC26A4* genotypes of patients are presented in Table 1. Thirteen different *SLC26A4* genotypes including recessive pathogenic *SLC26A4* variants were found in patients: four genotypes with homozygous variants, five genotypes with compound heterozygous variants, and four genotypes with single variants (Table 1).

In total, six different pathogenic or likely pathogenic *SLC26A4* variants were found in both cohorts of patients (Table 2). Among them, the variants c.170C>A (p.Ser57Ter), c.919-2A>G, c.2027T>A (p.Leu676Gln), c.2034+1G>A, and c.2168A>G (p.His723Arg) were previously found in patients with HL in different regions of the world while c.1545T>G (p.Phe515Leu) was a novel *SLC26A4* variant.

For patients who were homozygous or compound heterozygous for pathogenic *SLC26A4* variants (*n* = 66, comprising 62 Tuvinians and 4 Altaians), the genetic diagnosis “Hearing loss due to the presence of two recessive mutations in the *SLC26A4* gene” could be established. Thus, the pathogenic contribution of the *SLC26A4* gene to HL of patients, defined as the proportion of patients with biallelic recessive pathogenic *SLC26A4* variants among all enrolled Tuvinian and Altaian patients, could be estimated as 28.2% (62/220) and 4.3% (4/93), respectively. Only one recessive pathogenic *SLC26A4* allele was identified in 14 patients (13 Tuvinians and 1 Altaian) (Table 1).

**Table 1:** The *SLC26A4* genotypes in Tuvinian and Altaian patients.

*SLC26A4* Genotypes			Tuvinian Patents (*n* = 220)	Altaian Patents (*n* = 93)
Homozygotes				
1	c.[919-2A>G];[919-2A>G] p.[splice acceptor variant];[splice acceptor variant]	intronic region between exons 7 and 8	30	-
2	c.[2027T>A];[2027T>A] p.[Leu676Gln];[Leu676Gln]	exon 17	4	-
3	c.[2168A>G];[2168A>G] p.[His723Arg];[His723Arg]	exon 19	-	2
4	c.[170C>A];[170C>A] p.[Ser57Ter];[Ser57Ter]	exon 3	1	-
	**Total**		**35**	**2**
**Compound heterozygotes**				
5	c.[919-2A>G];[2027T>A] p.[splice acceptor variant];[Leu676Gln]	intronic region between exons 7 and 8/exon 17	14	2
6	c.[919-2A>G];[1545T>G] * p.[splice acceptor variant];[Phe515Leu] *	intronic region between exons 7 and 8/exon 14	8	-
7	c.[170C>A];[919-2A>G] p.[Ser57Ter];[splice acceptor variant]	exon 3/intronic region between exons 7 and 8	3	-
8	c.[919-2A>G];[2034+1G>A] p.[splice acceptor variant];[splice donor variant]	intronic region between exons 7 and 8/intronic region between exons 17 and 18	1	-
9	c.[1545T>G] *;[2027T>A] p.[Phe515Leu] *;[Leu676Gln]	exons 14/17	1	-
	**Total**		**27**	**2**
**Biallelic *SLC26A4* mutations in total**			**62 (28.2%)**	**4 (4.3%)**
**Single heterozygotes**				
10	c.[919-2A>G];[?] p.[splice acceptor variant];[?]	intronic region between exons 7 and 8	9	-
11	c.[1545T>G] *;[?] p.[Phe515Leu] *;[?]	exon 14	2	-
12	c.[170C>A];[?] p.[Ser57Ter];[?]	exon 3	1	-
13	c.[2027T>A];[?] p.[Leu676Gln];[?]	exon 17	1	1
	**Total**		**13 (5.9%)**	**1 (1.1%)**

The *SLC26A4* variations are designated at the nucleotide level (NC_000007.14, https://www.ncbi.nlm.nih.gov/nuccore/NC_000007.14/ (accessed on 1 November 2021) and amino acid level (NP_000432.1, https://www.ncbi.nlm.nih.gov/protein/NP_000432.1/ (accessed on 1 November 2021) at the top and bottom of each line, respectively. *—novel variant in the *SLC26A4* gene.

**Table 2:** Pathogenic variants in the *SLC26A4* gene found in Tuvinian and Altaian patients.

	SLC26A4 Variants		Location	Molecular Consequence	dbSNP ID	ClinVar (2021)
	Nucleotide	Amino Acid				
1	c.170C>A	p.Ser57Ter	exon 3	nonsense variant	rs111033200	pathogenic
2	c.919-2A>G	splice acceptor variant	intronic region between exons 7 and 8	splice acceptor	rs111033313	pathogenic
3	c.1545T>G *	p.Phe515Leu	exon 14	missense variant	not presented	not presented
4	c.2027T>A	p.Leu676Gln	exon 17	missense variant	rs111033318	pathogenic/likely pathogenic
5	c.2034+1G>A	splice donor variant	intronic region between exons 17 and 18	splice donor	rs759683649	likely pathogenic
6	c.2168A>G	p.His723Arg	exon 19	missense variant	rs121908362	pathogenic/likely pathogenic

*—novel variant in the *SLC26A4* gene.

**Figure 1:**
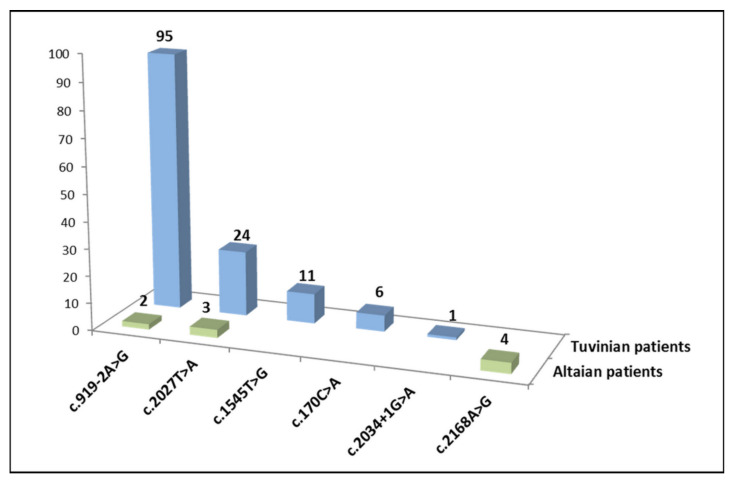
Distribution of pathogenic variants among all mutated *SLC26A4* alleles in Tuvinian and Altaian patients.

Variant c.919-2A>G was the most frequent of all pathogenic *SLC26A4* variants detected in Tuvinian patients (95/137, 69.3%), followed by c.2027T>A (p.Leu676Gln) (24/137, 17.5%), c.1545T>G (p.Phe515Leu) (11/137, 8.0%), c.170C>A (p.Ser57Ter) (6/137, 4.4%), and c.2034+1G>A (1/137, 0.7%). Variant c.2168A>G (p.His723Arg) was prevalent in Altaian patients (4/9, 44.5%) followed by c.2027T>A (p.Leu676Gln) (3/9, 33.3%), and c.919-2A>G (2/9, 22.2%) (Figure 1).

### 3.2. Novel SLC26A4 Variant c.1545T>G (p.Phe515Leu)

**Figure 2:**
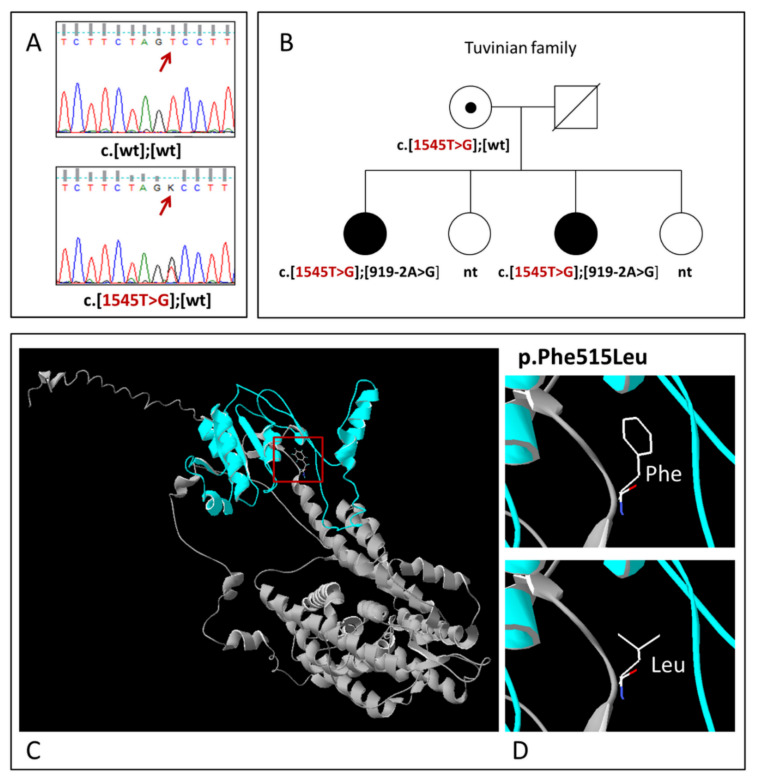
(**A**) Identification of variant c.1545T>G (p.Phe515Leu) by Sanger sequencing; (**B**) The pedigree of the Tuvinian family demonstrating the segregation of variant c.1545T>G (p.Phe515Leu) in compound with recessive mutation c.919-2A>G with HL. Deaf individuals are shown by black symbols; the variant c.1545T>G (p.Phe515Leu) is shown by red; nt—not tested; wt—wild-type; (**C**) The 3D structure of the pendrin protein with localization of variant p.Phe515Leu; (**D**) Close-up views of wild (Phe515) and mutant (Leu515) types of pendrin.

The c.1545T>G is a novel, previously undescribed, missense variant in exon 14 of *SLC26A4* leading to substitution of phenylalanine by leucine at amino acid position 515 (p.Phe515Leu) of the pendrin protein (Figure 2). This variant was found in 11 Tuvinian patients from 8 unrelated families: in 9 patients in a compound with already known *SLC26A4* mutations (c.919-2A>G or c.2027T>A) and in 2 patients in a heterozygous state (Table 1).

The analysis of available family members in one Tuvinian family where the c.1545T>G (p.Phe515Leu) variant was found revealed the segregation of c.1545T>G (p.Phe515Leu) with HL (Figure 2). Unfortunately, the testing of the relatives of other patients with this variant was not available to support strong segregation of c.1545T>G (p.Phe515Leu) with HL. In addition, the allelic frequency of c.1545T>G was estimated in the group of Tuvinian patients tested for c.1545T>G (137individuals) and in the Tuvinian control sample. To exclude possible bias in the estimation of c.1545T>G frequency in a group of patients owing to the presence of a certain number of related individuals, we used a sample of unrelated patients formed by analysis of their pedigrees (121 individuals, 242 alleles) for a comparative analysis. The frequency of c.1545T>G in this sample of Tuvinian patients (3.7%, 9/242) was significantly higher than in the Tuvinian control sample (1.0%, 3/296) (*p* = 0.03391). We also evaluated a potential functional significance of this novel variant using 11 bioinformatics predictive software tools. Most of them predicted a potentially deleterious effect (“damaging”/“disease causing”/“possibly damaging”) of this missense variant (Supplementary Table S2).

### 3.3. Carrier Frequency of Pathogenic SLC26A4 Variants in Tuvinian and Altaian Control Samples

Based on the prevalence of pathogenic *SLC26A4* variants in both groups of patients (Tuvinians and Altaians), we screened the most frequent pathogenic *SLC26A4* variants in appropriate ethnically matched control samples. Two pathogenic variants, c.919-2A>G and c.1545T>G, were found with frequencies of 5.1% (8/157) and 2.0% (3/148), respectively, among unrelated healthy Tuvinians, while none of the pathogenic *SLC26A4* variants were detected in the Altaian control sample (Table 3).

The allelic frequency of each pathogenic *SLC26A4* variant (except for the very rare c.2034+1G>A found in one Tuvinian patient) in both groups of patients (Tuvinians and Altaians) was significantly higher (*p* < 0.05) than in the corresponding ethnic controls. For a correct comparative analysis, the samples of unrelated patients were used.

### 3.4. Computed Tomography (CT) of the Temporal Bones in Tuvinian Patients

To elucidate the prevalence of the enlarged vestibular aqueduct (EVA) in Tuvinian patients homozygous or compound heterozygous for the *SLC26A4* mutations, the temporal bone computed tomography (CT) was performed. Unfortunately, the CT examination was available only for 27 out of 62 Tuvinian patients with biallelic *SLC26A4* mutations. These patients (15 females and 12 males, aged from 11 to 57 years old) belonged to 19 unrelated families. Clinical descriptions and CT medical reports of patients are presented in Supplementary Table S3. Among 27 patients who passed the CT examination, the genotype c.[919-2A>G];[919-2A>G] was prevalent (15 patients) followed by the genotype c.[919-2A>G];[2027T>A] (7 patients), and one of the other five genotypes (c.[919-2A>G];[1545T>G], c.[2027T>A];[2027T>A], c.[919-2A>G];[2034+1G>A], c.[170C>A];[170C>A] or c.[170C>A];[919-2A>G]) was found in single patients. The CT scans were interpreted by the specialists from the Department of Diagnostic Radiology of the Republican Hospital No. 1 (Kyzyl, the Tyva Republic, Russia) according to the most accepted “Valvassori” criterion for the definition of EVA [18]: a vestibular aqueduct was considered to be enlarged if its diameter was >1.5 mm at the midpoint between the common crus and the external aperture of the vestibular aqueduct on CT images. A total of 24 out of 27 examined patients had bilateral EVA varying from 1.5 to 5.1 mm; the vestibular aqueduct up to 1.5 mm in both ears was found in one patient; unilateral EVA was observed in two patients. The results showed that the degree of EVA in examined patients can differ in both ears of the same patient and is characterized by intrafamilial and interfamilial variability (Supplementary Table S3).

## 4. Discussion

### 4.1. The SLC26A4-Related HL in Tuvinian and Altaian Patients

In this study, we investigated the prevalence of the *SLC26A4* pathogenic variants in Tuvinian and Altaian patients where the causes of HL remained unknown after thorough testing for the *GJB2* gene [45,48,49,51] and target screening for several mutations in other genes (*MT-RNR1*, *MT-TS1*, *OTOF*, *RAI1*) [46,47,50]. Unlike most studies, in which the *SLC26A4* gene was generally tested in the cohorts of patients with already diagnosed EVA, the patients in our study were not preselected by the presence of EVA. This approach allowed us to estimate the overall pathogenic contribution of *SLC26A4* mutations in HL in total groups of Tuvinian (*n* = 220) and Altaian patients (*n* = 93). The presence of biallelic recessive pathogenic *SLC26A4* variants explained the etiology of HL (DFNB4) in 28.2% (62/220) of Tuvinian patients and in 4.3% (4/93) of Altaian patients (Table 1). To our knowledge, the rate of the *SLC26A4*-related HL in Tuvinian patients (28.2%) is one of the highest among populations worldwide. In addition, the significant difference in the prevalence of *SLC26A4*-caused HL (28.2% in Tuvinians vs. 4.3% in Altaians) among two neighboring indigenous Turkic-speaking Siberian peoples with a common ethnic background is an unexpected and interesting observation.

The enlarged vestibular aqueduct (EVA) detected by CT or MRI scanning is a specific feature of DFNB4 in the majority of patients with *SLC26A4* mutations. The EVA syndrome was first described in the study by Valvassori and Clemis (1978) where the vestibular aqueduct was considered as enlarged if its anteroposterior diameter was more than 1.5 mm in the midpoint of the post isthmic segment or halfway between the external aperture and the common crus [18]. Subsequently, these criteria were generally considered to be the defining characteristics of EVA in patients with HL. Based on a review of a pediatric HL database and the radiographic comparisons to a group of normal hearing children, Boston et al. (2007) and Vijayasekaran et al. (2007) proposed to define EVA as one that is 2 mm at the operculum and/or 1 mm at the midpoint [19,20]. Dewan et al. (2009) reported that the use of these criteria (referred to as “Cincinnati criteria”) allowed identification of a large percentage (significantly greater than would have been identified by the Valvassori criterion) of pediatric cochlear implant patients with EVA who might otherwise have no known etiology for their deafness [56]. Currently, there are no uniform and standardized criteria for the diagnosis of EVA, and both criteria (the Valvassori criteria or the Cincinnati criteria) are used in different studies.

In our study, the temporal bone computed tomography (CT) was performed only in a limited number of Tuvinian patients (27 out of 62 patients homozygous or compound heterozygous for the *SLC26A4* mutations) because of the unavailability of CT examination for patients living in small remote villages in various administrative districts of the Tyva Republic. According to the conventional Valvassori criterion, bilateral EVA (a midpoint diameter from 1.5 to 5.1 mm) was observed in the majority (24 out of 27) of examined patients with different *SLC26A4* genotypes. The EVA degree differed in both ears of the same patient and was characterized by intrafamilial and interfamilial variability (Supplementary Table S3). When these results were reviewed using the Cincinnati criteria, all patients appeared to have bilateral EVA, except one with unilateral EVA (patient #17, male, 18 years old, genotype c.[919-2A>G];[919-2A>G]) (Supplementary Table S3). These results were consistent with the abundant data confirming EVA in the vast majority of patients with biallelic *SLC26A4* mutations. Unfortunately, the limited number of CT-examined patients did not allow us to identify any correlations of the EVA degree with a certain *SLC26A4* genotype.

### 4.2. Pathogenic SLC26A4 Variants in Tuvinians and Altaians

A total of six different pathogenic *SLC26A4* variants were identified in patients in our study (Table 2). Two of them, c.919-2A>G and c.2168A>G (p.His723Arg), were the most frequent among Tuvinian and Altaian patients, respectively (Figure 1).

The proportions of c.919-2A>G among all mutant *SLC26A4* alleles identified were 69.9% in Tuvinian patients and 22.2% in Altaian patients. The c.919-2A>G mutation (previously named IVS7-2A>G, rs111033313) is located at the splice site in the intron region between exons 7 and 8 and leads to a skipping of exon 8, with the formation of a stop codon at amino acid position 311 and finally a truncated form of pendrin molecule. This mutation was firstly identified in an extended inbred Turkish family [57]. In numerous subsequent studies, c.919-2A>G was often detected in deaf subjects from Asian countries (mainland China, Taiwan, Mongolia, Korea, and Japan) and observed with the highest frequency in China [6,8,12,13,39,58].

The c.2168A>G (p.His723Arg, rs121908362) mutation, detected only in Altaian patients, was one of the first *SLC26A4* mutations identified in patients with Pendred syndrome and EVA [59,60]. Subsequently, c.2168A>G (p.His723Arg) was found to be the predominant *SLC26A4* mutation in patients from Japan and Korea [6,10,61].

Thus, c.919-2A>G and c.2168A>G (p.His723Arg) are thought to be the most common *SLC26A4* mutations in Asian populations. High frequencies of c.2168A>G (p.His723Arg) in Japanese and Koreans, and c.919-2A>G in Han Chinese (Taiwanese) are probably the result of the founder effect [6,62].

Variant c.2027T>A (rs111033318) in exon 17 of the *SLC26A4* gene results in substitution of leucine by glutamine at position 676 (p.Leu676Gln) in the pendrin amino acid sequence. This variant was predicted to disrupt an α-helical domain of pendrin leading to altered trafficking of pendrin and its intracellular retention [63,64]. Variant c.2027T>A (p.Leu676Gln) appears to be specific for Asian populations, since it was previously detected, although relatively rare, in patients from China, Mongolia, and Korea [6,8,12,31,32,65,66]. In our study, unlike the studies in China, Mongolia, and Korea, variant c.2027T>A (p.Leu676Gln) was found in a significant number of patients (19 Tuvinians and 2 Altaians) and was the second most frequent pathogenic *SLC26A4* variant in both cohorts of examined patients.

The variant c.170C>A (rs111033200) in exon 3 of the *SLC26A4* gene was detected in our study only in five Tuvinian patients. This mutation leads to the formation of a stop codon at amino acid position 57 (p.Ser57Ter) at the NH_2_-terminus of the pendrin molecule, and the protein is predicted to lack most of the important domains [67]. The c.170C>A (p.Ser57Ter) mutation was previously found in several deaf patients from India, China, Pakistan, Mexico, and Turkey [6,28,66,67,68].

Variant c.2034+1G>A was found in one Tuvinian patient in a compound heterozygous state with mutation c.919-2A>G. This mutation affects a donor splice site in intron 17 of the *SLC26A4* gene and has been classified as “likely pathogenic”, since it is expected to disrupt RNA splicing and likely to result in the disrupted protein product. This variant has not been reported in the literature in individuals with *SLC26A4*-related conditions and currently presents only in population databases (rs759683649, ExAC: 0.009%). Detection of c.2034+1G>A in a deaf patient in our study supported the pathogenicity of this variant but additional data are required to prove that conclusively.

The missense variant c.1545T>G (NC_000007.13:g.107338487 T>G, p.Phe515Leu) (Figure 2) in exon 14 of *SLC26A4* was found for the first time in Tuvinian patients and the Tuvinian control sample. Several lines of evidence (segregation of c.1545T>G with HL in affected subjects from several unrelated families; significantly higher frequency of this variant in patients compared with ethnically matched controls; multiple computational predictions of its deleterious effect; current absence in the world human genome databases) support the presumed pathogenicity of this variant. It is worth noting that two other rare *SLC26A4* variants, c.1544T>C (NC_000007.13:g.107336484T>C, rs138132962) and c.1544T>G (NC_000007.13:g.107336484T>G), leading to amino acid substitutions at the same position 515 (NP_000432.1:p.Phe515Ser and NP_000432.1:p.Phe515Cys, respectively), were characterized as “pathogenic” (DEAFNESS VARIATION DATABASE https://deafnessvariationdatabase.org/ (accessed on 1 November 2021). Both c.1544T>C and c.1544T>G in compound with other *SLC26A4* mutations were previously found in Chinese or Turkish patients, respectively [66,69].

In total, we revealed a relatively narrow spectrum of *SLC26A4* mutations in Tuvinian and Altaian patients, which was characterized by the presence of Asian-specific variants—c.919-2A>G (predominant in Tuvinians), c.2168A>G (p.His723Arg) (found only in Altaians), and also c.2027T>A (p.Leu676Gln), whose frequency in Tuvinians was significantly higher than in other populations worldwide. In addition, a high frequency of a novel, likely pathogenic, variant c.1545T>G (p.Phe515Leu) was observed in Tuvinian patients.

### 4.3. Comparative Analysis of Genetic Causes of HL in Tuvinian and Altaian Patients

**Figure 3:**
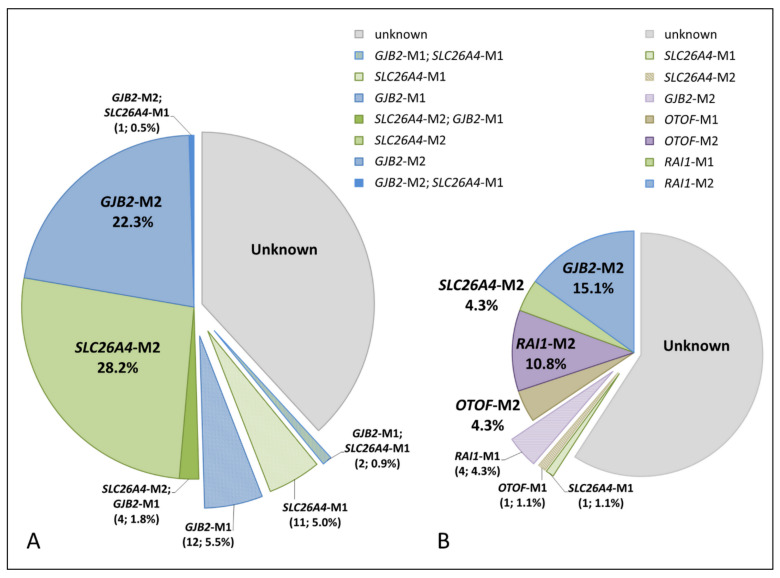
The genetic causes of HL in Tuvinian (**A**) and Altaian (**B**) patients. *SLC26A4*-M2 and *SLC26A4*-M1—biallelic and monoallelic *SLC26A4* mutations, respectively; *GJB2*-M2 and *GJB2*-M1—biallelic and monoallelic *GJB2* mutations, respectively; *RAI1*-M2 and *RAI1*-M1—biallelic and monoallelic mutation c.5254G>A (p.Gly1752Arg) in the *RAI1* gene, respectively; *OTOF*-M2 and *OTOF*-M1—biallelic and monoallelic mutation c.1111C>G (p.Gly371Arg) in the *OTOF* gene, respectively; unknown—no pathogenic variants were found in the studied genes. The pie chart area is proportional to the size of each examined group.

We compared the ascertained genetic causes of HL in Tuvinian and Altaian patients by combining the results of the *SLC26A4* analysis performed in this study with the data from our previous studies aimed at elucidating the genetic components of HL in these indigenous peoples of Southern Siberia [45,46,47,48,49,50,51]. In total, we revealed the genetic causes of HL in 50.5% of Tuvinian patients and in 34.5% of Altaian patients (Figure 3).

Along with 66 patients with biallelic *SLC26A4* genotypes, 14 patients (13 Tuvinians and 1 Altaian) were the carriers of a single recessive *SLC26A4* pathogenic variant (Table 1). Most of them had severe-to-profound HL. Our previous *GJB2* gene testing revealed biallelic *GJB2* mutations in 49 out of 220 Tuvinian patients (22.3%) while 18 (8.2%) Tuvinian patients appeared to be the coincidental carriers of one pathogenic *GJB2* allele [48]. When we compared the results of the *SLC26A4* testing in Tuvinian patients with their *GJB2* genotypes, four patients with the *SLC26A4*-related HL were also the carriers of one pathogenic *GJB2* allele and two patients were coincidently *GJB2-* and *SLC26A4*-heterozygotes. Moreover, additional *SLC26A4* testing revealed heterozygous *SLC26A4* variant c.1545T>G (p.Phe515Leu) in one Tuvinian patient with biallelic *GJB2* mutations (Figure 3).

A relatively large proportion of deaf individuals carrying only one recessive *SLC26A4* pathogenic variant has been reported in many studies [35,70,71,72], and diagnostic interpretation in such cases remains problematical. Several assumptions have been made to resolve this issue: HL in these patients could be caused by an uncertain impact of the *SLC26A4* gene (the presence of yet undetected regulatory or deep-intronic variants and intragenic exon deletions); HL could be the result of digenic inheritance; these patients could be only the coincidental carriers of one pathogenic *SLC26A4* variant and, consequently, other factors (other genes or environmental impacts) caused their HL.

A thorough analysis of all 21 exons and adjacent regions in *SLC26A4* did not reveal any other pathogenic variants in our *SLC26A4* monoallelic patients. There were no statistically significant differences in the frequency of monoallelic *SLC26A4* mutations among Tuvinian patients in whom two pathogenic *SLC26A4* mutations were not identified, compared to the Tuvinian control sample (data not shown). Thus, although we cannot completely rule out any unrecognized *SLC26A4* variants in other regions of the *SLC26A4* sequence or large deletions, the *SLC26A4* monoallelic patients in our samples were more likely to be coincidental carriers of a single *SLC26A4* pathogenic variant, and other factors (other genes or environmental impacts) could have caused their HL.

## 5. Conclusions

In conclusion, thorough testing of the *SLC26A4* gene is essential for establishing a genetic diagnosis of HL in the indigenous populations of Southern Siberia. The data obtained in this study provide important targeted information for genetic counseling of affected Tuvinian and Altaian families and enrich the current information on the *SLC26A4* gene variability worldwide.

## Figures and Tables

**Table 3:** The carrier frequency of pathogenic *SLC26A4* variants in Tuvinian and Altaian control samples.

Pathogenic *SLC26A4* Variants	Tuvinian Control Sample	Altaian Control Sample
c.919-2A>G	5.1% (8/157)	nt
c.1545T>G	2.0% (3/148)	nt
c.170C>A	0% (0/100)	nt
c.2027T>A	0% (0/157)	0% (0/123)
c.2034+1G>A	0% (0/157)	0% (0/123)
c.2168A>G	nt	0% (0/141)

nt—not tested.

## Data Availability

The data presented in this study are available in this article and Supplementary Materials.

## Supplementary Materials

The following are available online at https://www.mdpi.com/article/10.3390/diagnostics11122378/s1, Table S1: Primers for PCR/Sanger sequencing and PCR-RFLP assays. Table S2: Bioinformatic predictions of the functional significance of a novel missense variant c.1545T>G (p.Phe515Leu) in the *SLC26A4* gene. Table S3: Clinical descriptions of Tuvinian patients with biallelic *SLC26A4* mutations.
