# Supplementary material for: Different Rates of the SLC26A4-Related Hearing Loss in Two Indigenous Peoples of Southern Siberia (Russia)
Source: Diagnostics (Basel). 2021 Dec 17;11(12):2378. doi: 10.3390/diagnostics11122378 (PMC8699871; doi:10.3390/diagnostics11122378)
Supplement: Supplementary file 1 [file diagnostics-11-02378-s001.zip › Supplementary Materials/Table S1.pdf]

**Table S1.** Primers for PCR / Sanger sequencing and PCR-RFLP assays.

| Exons | Primers | Size of fragment (bp) | Reference |
|---|---|---|---|
| **PCR / Sanger sequencing** | | | |
| Exon 1 | PDS_Ex1-3_F: ACTCATTCACTTCTCAGGAAAACG<br>sqPDS_Ex1R: CTCGTCCTGTTTTCCGAGCA | 1208 | This study |
| Exons 2-3 | sqPDS_Ex2F: TCGTCCTCGCTTACCGCGTGT<br>sqPDS_Ex3R: CCATCTGTCACAGACCTATGGTAGCT | 1932 | This study |
| Exon 4 | sqPDS_Ex4F: AGGCTATCCTACCTGAAATGTGCT<br>sqPDS_Ex4R: GAAATCCCATTTCCCTGACAACA | 608 | This study |
| Exons 5-6 | sqPDS_Ex5F: CTATGCAGACACATTGAACATTTGT<br>SLC26A4_Ex6R: ATTGTTTCTGGAATGAACAGTGACC | 1065 | This study<br>[Everett et al., 1997] |
| Exons 7-8 | PDS_Ex7-8_F: CATGGTTTTTCATGTGGGAAGATTC<br>PDS_Ex7-8_R: CAAATGGCTTGACGTTTATCTACACAC | 636 | [Everett et al., 1997] |
| Exons 9-10 | sqPDS_Ex9F: GAGCAGATATAGCATTTGATGAGATG<br>sqPDS_Ex10R: CTCGGTGCGAGCCTTCCT | 1343 | This study |
| Exons 11-12 | sqPDS_Ex11F: CGAAGTGTTATCAGAGTTGCTATTATAG<br>sqPDS_Ex12R: GCATTGTTTTGTATGTTGTTTGTCC | 1119 | This study |
| Exon 13 | sqPDS_Ex13F: CGGGTTTGGTGGTATGTACTTGT<br>sqPDS_Ex13R: ACAGCAGTAGAGGACATGAGTAGGTAC | 696 | This study |
| Exon 14 | sqPDS_Ex14F: TCCAGGCCTTTTCTCACTTCTC<br>sqPDS_Ex14R: TCTTTGTTTCCCCTAGTCACCACT | 1286 | This study |
| Exons 15-16 | sqPDS_Ex15F: AGTTGAGTGCTGCTACCCAGC<br>sqPDS_Ex16R: CCACTCCCGCTTGCCTATAAG | 1408 | This study |
| Exon 17 | sqPDS_Ex17F: GCAAAGTTCCACAATCATCCAGA<br>sqPDS_Ex17R: TGTTGCAATACTGGACAACCCA | 544 | This study |
| Exon 18 | SLC26A4_EX18F: GCTGGATGTTGCCATCTCTTGAG<br>sqPDS_Ex18R: CTAAGCTCCCGCACTAGACGA | 835 | [Everett et al., 1997]<br>This study |
| Exon 19 | sqPDS_Ex19F: TCGTCCACAAGGTTGACTACGAC<br>sqPDS_Ex19R: GGCATTATAGCTAATGCCCACT | 811 | This study |
| Exon 20 | sqPDS_Ex20F: GAATAACCAACTTGGTCCATAGACA<br>sqPDS_Ex20R: AGAATCAGTCTAGGGTGGGGAGA | 1010 | This study |
| Exon 21 | SLC26A4_Ex21F: CTGGGCAACAGTGAGTGAGA<br>SLC26A4_Ex21R: GCATTGAGGAAGTTTTGTCTTG | 296 | [Prasad et al., 2004] |
| **PCR-RFLP assays** | | | |
| Detection of c.170C>A (*Tru9 I*) | 170C>A-F: ACTTCAGGGTTATTATTTTCCA<br>170C>A R: AGCCATTCCTTGACTCGGT | 234 | This study |
| Detection of c.919-2A>G (*Hpa II*) | 919-2A-F: TGGAGTTTTTAACATCTTTTGTTTTATTCC<br>919-2A-R: TGCCATGTTTTAAGAAGAAAATGAAGAA | 354 | [Dai et al., 2008]<br>This study |
| Detection of c.1545T>G (*Pce I*) | sqPDS_Ex14F: TCCAGGCCTTTTCTCACTTCTC<br>sqPDS_Ex14R: TCTTTGTTTCCCCTAGTCACCACT | 1286 | This study |
| Detection of c.2168A>G (*Rsr2 I*) | Ex19_F: CGTGATCGTCCACAAG<br>Ex19_R: GGGTACTACCAGGTAATTT | 754 | This study |

**References for Table S1**

Everett, L.A.; Glaser, B.; Beck, J.C.; Idol, J.R.; Buchs, A.; Heyman, M.; Adawi, F.; Hazani, E.; Nassir, E.; Baxevanis, A.D.; et al. Pendred syndrome is caused by mutations in a putative sulphate transporter gene (PDS). *Nat. Genet*. **1997**, *17*, 411–422, doi: 10.1038/ng1297-411.

Dai, P.; Li, Q.; Huang, D.; Yuan, Y.; Kang, D.; Miller, D.T.; Shao, H.; Zhu, Q.; He, J.; Yu, F.; et al. *SLC26A4* c.919-2A>G varies among Chinese ethnic groups as a cause of hearing loss. *Genet. Med*. **2008**, *10*, 586–592, doi: 10.1097/gim.0b013e31817d2ef1.

Prasad, S.; Kölln, K.A.; Cucci, R.A.; Trembath, R.C.; van Camp, G.; Smith, R.J. Pendred syndrome and DFNB4-mutation screening of *SLC26A4* by denaturing high-performance liquid chromatography and the identification of eleven novel mutations. *Am. J. Med. Genet. A*. **2004**, *124A*, 1–9, doi: 10.1002/ajmg.a.20272.
