# Supplementary material for: Different Rates of the SLC26A4-Related Hearing Loss in Two Indigenous Peoples of Southern Siberia (Russia)
Source: Diagnostics (Basel). 2021 Dec 17;11(12):2378. doi: 10.3390/diagnostics11122378 (PMC8699871; doi:10.3390/diagnostics11122378)
Supplement: Supplementary file 1 [file diagnostics-11-02378-s001.zip › Supplementary Materials/Table S2.pdf]

Table S2. Bioinformatic predictions of the functional significance of a novel missense variant c.1545T>G (p.Phe515Leu) in the *SLC26A4* gene.

Prediction software tools	Deleterious threshold	Prediction (Scores)	References
**FATHMM** (http://fathmm.biocompute.org.uk/)	< - 1.5	Damaging (- 3.16)	[Shihab et al., 2013]
**Mutation Taster** (http://www.mutationtaster.org/)	> 0.5	Disease Causing (0.99)	[Schwarz et al., 2014]
**PROVEAN** (http://provean.jcvi.org)	< - 2.5	Damaging (- 4.20)	[Choi et al., 2012; Choi et al., 2015]
**PolyPhen-2** (http://genetics.bwh.harvard.edu/pph2)	> 0.85	Possibly damaging (0.849)	[Adzhubei et al., 2010]
**MutPred2** (http://mutpred.mutdb.org/)	≥ 0.67	Possibly damaging (0.54)	[Pejaver et al., 2020]
**Condel** (https://bbglab.irbbarcelona.org/fannsdb/)	> 0.49	Damaging (0.55)	[González-Pérez et al., 2011]
**SNPs&GO** (https://snps-and-go.biocomp.unibo.it/snps-and-go/)	-	Disease-related Polymorphism	[Calabrese et al., 2009]
**CADD** (https://cadd.gs.washington.edu/)	> 15	21.8	[Rentzsch et al., 2018]
**Align-GVGD** (http://agvgd.hci.utah.edu/)	> C65	Low (C15)	[Tavtigian et al., 2005; Mathe et al., 2006]
**MutationAssessor.org** (http://mutationassessor.org/)	> 3.5	Low (1.19)	[Reva et al., 2007; Reva et al., 2011]
**SIFT** (https://sift.bii.a-star.edu.sg/)	< 0.05	Tolerated (0.33)	[Sim et al., 2012]

References for Table S2

Adzhubei, I.A.; Schmidt, S.; Peshkin, L.; Ramensky, V.E.; Gerasimova, A.; Bork, P.; Kondrashov, A.S.; Sunyaev, S.R. A method and server for predicting damaging missense mutations. *Nat. Methods* **2010**, *7*, 248–249, doi: 10.1038/nmeth0410-248.

Calabrese, R.; Capriotti, E.; Fariselli, P.; Martelli, P.L.; Casadio, R. Functional annotations improve the predictive score of human disease-related mutations in proteins. *Hum. Mutat*. **2009**, *30*, 1237–1244, doi: 10.1002/humu.21047.

Choi, Y.; Sims, G.E.; Murphy, S.; Miller, J.R.; Chan, A.P. Predicting the functional effect of amino acid substitutions and indels. *PLoS One* **2012**, *7*, e46688, doi: 10.1371/journal.pone.0046688.

Choi, Y.; Chan, A.P. PROVEAN web server: A tool to predict the functional effect of amino acid substitutions and indels. *Bioinformatics* **2015**, *31*, 2745–2747, doi: 10.1093/bioinformatics/btv195.

González-Pérez, A.; López-Bigas, N. Improving the assessment of the outcome of nonsynonymous SNVs with a consensus deleteriousness score, Condel. *Am. J. Hum. Genet*. **2011**, *88*, 440–449, doi: 10.1016/j.ajhg.2011.03.004.

Mathe, E.; Olivier, M.; Kato, S.; Ishioka, C.; Hainaut, P.; Tavtigian, S.V. Computational approaches for predicting the biological effect of p53 missense mutations: a comparison of three sequence analysis based methods. *Nucleic. Acids Res*. **2006**, *34*, 1317–1325, doi: 10.1093/nar/gkj518.

Pejaver, V.; Urresti, J.; Lugo-Martinez, J.; Pagel, K.A.; Lin, G.N.; Nam, H.J.; Mort, M.; Cooper, D.N.; Sebat, J.; Iakoucheva, L.M.; et al. Inferring the molecular and phenotypic impact of amino acid variants with MutPred2. *Nat. Commun*. **2020**, *11*, 5918, doi: 10.1038/s41467-020-19669-x.

Rentzsch, P.; Witten, D.; Cooper, G.M.; Shendure, J.; Kircher, M. CADD: predicting the deleteriousness of variants throughout the human genome. *Nucleic. Acids Res.* **2019**, *47(D1)*, D886–D894. doi: 10.1093/nar/gky1016

Reva, B.; Antipin, Y.; Sander, C. Determinants of protein function revealed by combinatorial entropy optimization. *Genome Biol*. **2007**, *8*, R232, doi: 10.1186/gb-2007-8-11-r232.

Reva, B.; Antipin, Y.; Sander, C. Predicting the functional impact of protein mutations: application to cancer genomics. *Nucleic. Acids Res.* **2011**, *39*,e118, doi: 10.1093/nar/gkr407.

Schwarz, J.M.; Cooper, D.N.; Schuelke, M.; Seelow, D. MutationTaster2: Mutation prediction for the deep-sequencing age. *Nat. Methods* **2014**, *11*, 361–362, doi: 10.1038/nmeth.2890.

Shihab, H.A.; Gough, J.; Cooper, D.N.; Stenson, P.D.; Barker, G.L.A.; Edwards, K.J.; Day, I.N.M.; Gaunt, T.R. Predicting the functional, molecular, and phenotypic consequences of amino acid substitutions using hidden markov models. *Hum. Mutat.* **2013**, *34*, 57–65, doi: 10.1002/humu.22225.

Sim, N.L.; Kumar, P.; Hu, J.; Henikoff, S.; Schneider, G.; Ng, P.C. SIFT web server: predicting effects of amino acid substitutions on proteins. *Nucleic. Acids Res.* **2012**, *40* (Web Server issue), W452–457, doi: 10.1093/nar/gks539.

Tavtigian, S.V.; Deffenbaugh, A.M.; Yin, L.; Judkins, T.; Scholl, T.; Samollow, P.B.; de Silva, D.; Zharkikh, A.; Thomas, A. Comprehensive statistical study of 452 BRCA1 missense substitutions with classification of eight recurrent substitutions as neutral. *J. Med. Genet*. **2006**, *43*, 295–305, doi: 10.1136/jmg.2005.033878.
