# Supplementary material for: Different Rates of the SLC26A4-Related Hearing Loss in Two Indigenous Peoples of Southern Siberia (Russia)
Source: Diagnostics (Basel). 2021 Dec 17;11(12):2378. doi: 10.3390/diagnostics11122378 (PMC8699871; doi:10.3390/diagnostics11122378)
Supplement: Supplementary file 1 [file diagnostics-11-02378-s001.zip › Supplementary Materials/Table S3.pdf]

Table S3. Clinical descriptions of Tuvinian patients with biallelic *SLC26A4* mutations.

**Unrelated families**	**Patients**	**Sex**	**Age ***	**Hl onset**	**Severity of HL**	***SLC26A4* genotypes**	**Enlarged vestibular aqueduct (EVA) (≥ 1.5 mm)**		
							**Right ear**	**Left ear**	**CT medical records**
Family #1	Patient #1	m	50	Early childhood	P	c.[919-2A>G];[919-2A>G]	< 1.5	< 1.5	CT-scan without pathology
	Patient #2	f	54	Congenital	P	c.[919-2A>G];[919-2A>G]	3.5	2.5	Bilateral EVA
	Patient #3	f	27	Congenital	P	c.[919-2A>G];[919-2A>G]	3.5	3.0	Bilateral EVA
Family #2	Patient #4	f	15	Congenital	P	c.[919-2A>G];[2027T>A]	2.3	2.8	Bilateral EVA
	Patient #5	f	14	Congenital	P	c.[919-2A>G];[2027T>A]	2.4	2.0	Bilateral EVA
	Patient #6	m	11	Congenital	P	c.[919-2A>G];[2027T>A]	2.0	2.3	Bilateral EVA
Family #3	Patient #7	m	24	Congenital	P	c.[919-2A>G];[919-2A>G]	3.1	2.8	Bilateral EVA
	Patient #8	f	32	Congenital	P	c.[919-2A>G];[919-2A>G]	2.0	1.7	Bilateral EVA
Family #4	Patient #9	f	20	Early childhood	P	c.[919-2A>G];[919-2A>G]	2.4	5.1	Bilateral EVA
	Patient #10	f	49	Congenital	P	c.[919-2A>G];[919-2A>G]	3.1	2.9	Bilateral EVA
	Patient #11	m	43	Congenital	P	c.[919-2A>G];[919-2A>G]	1.8	1.9	Bilateral EVA
Family #5	Patient #12	f	53	Congenital	P	c.[919-2A>G];[1545T>G]	3.8	3.2	Bilateral EVA
	Patient #13	f	32	Congenital	P	c.[919-2A>G];[2027T>A]	3.2	2.7	Bilateral EVA
Family #6	Patient #14	m	11	Congenital	P	c.[919-2A>G];[2034+1G>A]	2.9	1.5	Bilateral EVA
Family #7	Patient #15	f	37	Congenital	P	c.[919-2A>G];[919-2A>G]	2.5	1.9	Bilateral EVA
Family #8	Patient #16	f	32	Early childhood	P	c.[2027T>A];[2027T>A]	2.0	1.9	Bilateral EVA
Family #9	Patient #17	m	18	Congenital	P	c.[919-2A>G];[919-2A>G]	2.0	0.9	Unilateral (right) EVA
Family #10	Patient #18	f	25	Congenital	P	c.[919-2A>G];[2027T>A]	3.1	2.4	Bilateral EVA
Family #11	Patient #19	m	13	Early childhood	P	c.[919-2A>G];[2027T>A]	3.5	3.2	Bilateral EVA
Family #12	Patient #20	m	28	Congenital	P	c.[919-2A>G];[919-2A>G]	2.5	1.3	Unilateral (left) EVA
Family #13	Patient #21	f	46	Early childhood	P	c.[919-2A>G];[2027T>A]	2.3	1.9	Bilateral EVA
Family #14	Patient #22	m	57	Early childhood	P	c.[919-2A>G];[919-2A>G]	3.1	1.5	Bilateral EVA
Family #15	Patient #23	m	28	Early childhood	P	c.[919-2A>G];[919-2A>G]	2.8	1.5	Bilateral EVA
Family #16	Patient #24	f	27	Early childhood	P	c.[170C>A];[170C>A]	5.0	3.9	Bilateral EVA
Family #17	Patient #25	m	19	Early childhood	P	c.[170C>A];[919-2A>G]	3.1	2.1	Bilateral EVA
Family #18	Patient #26	f	16	Early childhood	P	c.[919-2A>G];[919-2A>G]	3.1	2.0	Bilateral EVA
Family #19	Patient #27	m	16	Early childhood	P	c.[919-2A>G];[919-2A>G]	2.6	1.7	Bilateral EVA

* – Age at the time of examination. HL – hearing loss; early childhood – under 3 years old; P – profound hearing loss.
